# Supine transfer test-induced changes in cardiac index predict fluid responsiveness in patients without intra-abdominal hypertension

**DOI:** 10.1186/s12871-023-02280-0

**Published:** 2023-09-18

**Authors:** Zhiyong Zhao, Zhongwei Zhang, Jing Liu, Zhili Xia, Qian Xing, Yaodong Zhang, Yijun Zheng, Lihua Shen, Qionghua Lin, Danyan Gu, Pengmei Wang, Shan Zhang, Fangfang Li, Biao Zhu

**Affiliations:** 1 Department of Critical Care, Fudan University Shanghai Cancer Center, Shanghai, 200032 China; 2 Department of Oncology, Shanghai Medical College, Fudan University, Shanghai, 200032 China

**Keywords:** Fluid responsiveness, Supine transfer, Cardiac index, Intra-abdominal hypertension

## Abstract

**Background:**

The reversible maneuver that mimics the fluid challenge is a widely used test for evaluating volume responsiveness. However, passive leg raising (PLR) does have certain limitations. The aim of the study is to determine whether the supine transfer test could predict fluid responsiveness in adult patients with acute circulatory failure who do not have intra-abdominal hypertension, by measuring changes in cardiac index (CI).

**Methods:**

Single-center, prospective clinical study in a 25-bed surgery intensive care unit at the Fudan University Shanghai Cancer Center**.** Thirty-four patients who presented with acute circulatory failure and were scheduled for fluid therapy. Every patient underwent supine transfer test and fluid challenge with 500 mL saline for 15–30 min. There were four sequential steps in the protocol: (1) baseline-1: a semi-recumbent position with the head of the bed raised to 45°; (2) supine transfer test: patients were transferred from the 45° semi-recumbent position to the strict supine position; (3) baseline-2: return to baseline-1 position; and (4) fluid challenge: administration of 500 mL saline for 15–30 min. Hemodynamic parameters were recorded at each step with arterial pulse contour analysis (ProAQT/Pulsioflex). A fluid responder was defined as an increase in CI ≥ 15% after fluid challenge. The receiver operating characteristic curve and gray zone were defined for CI.

**Results:**

Seventeen patients were fluid challenge. The *r* value of the linear correlations was 0.73 between the supine transfer test- and fluid challenge-induced relative CI changes. The relative changes in CI induced by supine transfer in predicting fluid responsiveness had an area under the receiver operating characteristic curve of 0.88 (95% confidence interval 0.72–0.97) and predicted a fluid responder with 76.5% (95% confidence interval 50.1–93.2) sensitivity and 88.2% (95% confidence interval 63.6–98.5) specificity, at a best threshold of 5.5%. Nineteen (55%) patients were in the gray zone (CI ranging from -3 and 8 L/min/m^2^).

**Conclusion:**

The supine transfer test can potentially assist in detecting fluid responsiveness in patients with acute circulatory failure without intra-abdominal hypertension. Nevertheless, the small threshold and the 55% gray zone were noteworthy limitation.

**Trial registration:**

Predicting fluid responsiveness with supine transition test (ChiCTR2200058264). Registered 2022–04-04 and last refreshed on 2023–03-26, https://www.chictr.org.cn/showproj.html?proj=166175.

**Supplementary Information:**

The online version contains supplementary material available at 10.1186/s12871-023-02280-0.

## Background

Fluid administration is a cornerstone of treatment in critically ill patients. Fluid challenge is the gold standard for assessing fluid responsiveness [1, 2]; however, may increase the risk of fluid overload [3, 4]. Pulse pressure variation (PPV) and stroke volume variation are commonly employed for fluid responsiveness detection; however, they do possess various limitations [5]. Mini-fluid challenge test (i.e. fluid challenge with 100ml of fluid) could be an alternative as well, with about 5% increase in fluid responders [6], but may leading to a false negative response [3]. Employing a reversible maneuver that mimics the fluid challenge to evaluate volume responsiveness is a possible strategy for minimizing this risk.

Passive leg raising (PLR) mimics a fluid challenge in which blood is transferred from the splanchnic vasculature and lower limbs to the right heart [7]. However, PLR has inherent limitations. Contraindications for PLR include lower extremity venous thrombosis, lower extremity atrophy, or patients who wear compression socks [8]. The supine transfer test involved transferring the patient from a semi-recumbent position (45°) to a strict supine position [7] to transfer blood from the splanchnic reservoir to the cardiac chambers but not from the lower extremities. To the best of our knowledge, two studies have described the hemodynamic effects of the supine transfer test [7, 9]. They reported that the cardiac index (CI) increased by approximately 4–5% during the test, but did not confirm if the supine transfer test could predict fluid challenge.

Arterial pulse contour analysis (ProAQT/Pulsioflex) is a dynamic and fast-response method used to measure CI in real-time. It is particularly suitable for measuring the response of a fluid-challenge when compared to other methods (i.e., pulmonary artery catheter, Echo, etc.). While the device may off less accurate CI measurements compared to transpulmonary thermodiltion [10], it can still be utilized to evaluate rapid and small changes in hemodynamic variables and determine the best treatment [11], for instance, in a mini-fluid challenge and end-expiratory occlusion test [12, 13] (https://doi.org/10.1186/s13054-019-2545-z).

This study aimed to determine whether the supine transfer test can predict fluid responsiveness by measuring changes in the CI using uncalibrated arterial pulse contour analysis in patients without intra-abdominal hypertension.

## Patients and methods

### Patients

This single-center, prospective clinical study was conducted in a 25-bed surgery intensive care unit (ICU). It was performed in accordance with the Declaration of Helsinki. The study was approved by the institutional review board of our hospital. All patients that were enrolled in the study provided written informed consent approved the study (No. 2203252–9-2303A, 2023.03.16). For patient who is unable to provide consent, informed consent is obtained from the authorized relatives. The study was registered in ChiCTR.gov (ChiCTR2200058264).

Inclusion criteria were as follows: (1) All adult patients (> 18 years) equipped with radial arterial catheter, (2) presented with acute circulatory failure with a systolic blood pressure below 90 mmHg, or experienced a decrease of more than 40 mmHg who previously hypertensive, (3) presented with other signs of acute circulatory failure (e.g., oliguria, skin mottling, tachycardia, hyperlactatemia).

Patients with arrhythmia, hip pain and mobility deficits, intra-abdominal hypertension (sustainably elevated intra-abdominal pressure (IAP) > 12 mmHg measured by intrabladder pressure [14], pulmonary hypertension, severe heart valve disease, thoracic aortic abnormalities, head trauma, and severe heart failure (heart failure progresses to stage D) [15] were excluded.

### Intervention

Every patient underwent a supine transfer test and fluid challenge with 500 mL saline for 20 min, as previously described [7]. To avoid misleading interpretations of CI changes during the supine transfer test and fluid challenge, precautions were taken to prevent adrenergic stimulation (induced by pain, coughing, discomfort, and awakening). The bed was adjusted from a semi-recumbent position (45°) in the supine position to perform the supine transfer. Bronchial secretions were carefully aspirated before the supine transfer test. When awake, patients were informed of the test [16]. Those participating in the study were required to have already been fitted with a radial arterial catheter line and central venous catheter located in the superior vena cava territory, enabling connection with the ProAQT/Pulsiflex equipment (Pulsion Medicical systems, Germany) and measurement of central venous pressure (CVP), respectively.

### Data collection

Information on the demographic and clinical characteristics, mechanical ventilation and its parameters, and dosage and use of vasopressors were collected. Hemodynamic variables at each step were also collected.

### Protocol

The protocol consisted of four sequential steps (Additional file 1: Figure E1): (1) Baseline-1: a semi-recumbent position with the head of the bed raised to 45°; (2) Supine transfer test: the patients were transferred from a 45° semi-recumbent position to a strict supine position; (3) Baseline-2: return to the baseline-1 position; (4) Fluid challenge: patients were administered of 500 mL of saline for 20 min.

For baseline-1 and baseline-2, the patient was stabilized for 2 min before hemodynamic variables were collected. In the second step (supine transfer test), the variables were collected when the CI reached the maximal value during the supine transfer test. Finally, the last step (fluid challenge) was performed immediately after the fluid challenge. For each enrolled patient, data on the heart rate, CVP, CI, PPV, systolic blood pressure (SBP), and diastolic blood pressure (DBP) were collected.

Following the protocol, patients were considered as fluid responders or non-responders. Fluid responders were defined as those with an increase in CI ≥ 15% after a fluid challenge [2, 17]; otherwise, they were classified as non-responders. During the study protocol, vasoactive/inotropic drugs or mechanical ventilation parameters were not changed.

### Hemodynamic monitoring

All patients were equipped with a special transducer, ProAQT/Pulsioflex, for CI through a radial arterial catheter. The initial CI value was estimated using a proprietary algorithm through an “autocalibration” with ProAQT/Pulsioflex, and values from the next steps were determined by pulse contour analysis with ProAQT/Pulsioflex. The data displayed by proAQT/Pulsioflex system is averaged over a 12-s rolling period. We track short-term changes in CI, and the data is automatically displayed by devices after processing, filtering, and averaging. The average of three consecutive measurements was used for analysis.

The central venous pressure is assessed by inserting a central venous catheter through the internal jugular veins. Monitoring of the central venous pressure can be done using a pressure transducer. The pressure transducer will be securely placed near the tricuspid valve on the right chest wall, and should be calibrated to atmospheric pressure before collecting the data [18].

The arterial pressure was calibrated using a 5-step approach [19]. After placing the arterial catheter, the pressure transducer needs to be leveled and zeroed, similar to monitoring the central venous pressure. Subsequently, we will assess the quality of the arterial blood pressure waveform to ensure the accuracy of blood pressure measurements.

### Statistical analysis

MedCalc Statistical Software (MedCalc Software bvba, Ostend, Belgium; https://www.medcalc.org; 2019) was used to estimate the sample size. As per the null hypothesis at 0.50, assuming an α error of 0.05, power of 0.9, and allocation ratio of 1, the area under the receiver operating characteristic (ROC) curve (AUC) was expected to be 0.80. Seventeen fluid responders and 17 nonresponders were included in this study.

The Kolmogorov–Smirnov test was performed to determine whether the data was normally distributed. Data are presented as mean (standard deviation), median (interquartile range), or number (frequency in %), as appropriate.

Continuous variables were evaluated between groups using the Wilcoxon or Friedman rank-sum test, whereas the Bonferroni post-hoc test was used for multiple pairwise comparisons. The Fisher’s exact test was used to compare categorical variables. The Pearson method was used to test linear correlations between the percent change in CI induced by the supine transfer test and fluid challenge.

The supine transfer-induced changes in continuous variables (CI, PPV, SBP, and DBP) and ROC curves were calcualted. The diagnostic performances of the tests were assessed by calculating the AUC [20]. The 95% confidence interval for the AUC was computed using the Delong method [21].

The gray zone approach establishes a range of values where no conclusion can be made regarding the potential responsiveness to fluid [22]. The best threshold for an AUC curve was defined as that which maximizes the Youden index (sensitivity + specificity -1) [23], the CIs for optimal cutoffs were computed using the gray zone approach (area of uncertainty of optimal cutoffs) [24, 25]. The response to each test below the lower or above the higher border of the gray zone were considered negative and positive, respectively. Responses within the gray zone were considered inconclusive.

A two-tailed *P* < 0.05 is considered to be statistically significant. Statistical analysis was performed using the MedCalc Statistical Software version 19.0.4.

## Results

### Study population

From April 2023 to June 2023, thirty-four patients were consecutively enrolled in this study (Additional file 1: Figure E2). All patients were included once during the study period. The patient characteristics are shown in the Additional file 1: Table E1. Based on the response of the CI to fluid challenge, 17 patients were considered responders. The mean age of all patients was 62 ± 11 years. The Acute Physiology and Chronic Health Evaluation II score on admission was 9 (7–11). The ICU mortality rate was 3%. At inclusion, six patients were mechanically ventilated with a tidal volume of 7.1 (6.6–7.6) mL/kg predicted body weight, positive end-expiratory pressure of 5 (5–5) cmH_2_O, and a plateau pressure of 18 (17–18) cmH_2_O. These patients were mechanically ventilated throughout the study protocol. Eight (24%) patients received norepinephrine at a dose of 0.30 (0.18–0.33) µg/kg/min. The results also showed that there were more hypotensive and tachycardia patients in the responders group than in the non-responders group, even though the *p*-value was greater than 0.05 in both groups.

### Baseline

**Fig. 1:**
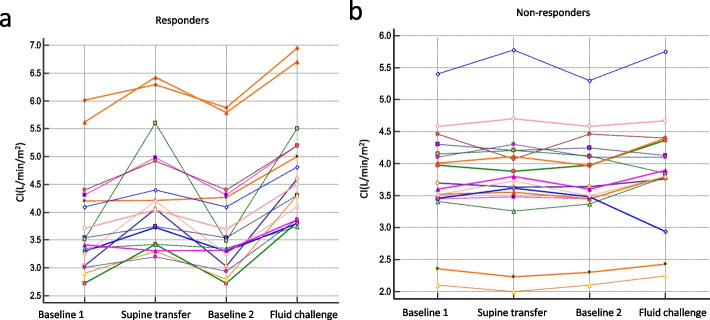
Individual values of cardiac index (CI) (**a**, **b**) in each step of the responders and non-responders

Baseline hemodynamic variables are presented in Table 1 and Fig. 1. None of the variables measured after returning to baseline-2 differed significantly from baseline-1.

**Table 1:** Hemodynamic variables at different study steps

Variable	Baseline 1	Supine Transfer	Baseline 2	Fluid Challenge
HR (beats/min)				
Responders	96(88–114)	97(85–113)	96(88–116)	95(82–109)
Non-responders	89(72–100)	90(73–97)	89(73–102)	90(73–95)
CVP (mmHg)				
Responders	7 (6–7)	9 (8–10)^b^	6 (6–8)	8(7–9)^b c^
Non-responders	7 (6–8)	8 (7–9)^b^	7 (6–8)	8 (7–9)^b^
SBP (mmHg)				
Responders	111(94–121)	113(100–119)	108(94–121)	114(101–122)^b c^
Non-responders	110(102–115)	109(105–116)	109(102–115)	113(109–121)^b c^
DBP (mmHg)				
Responders	52(45–61)	54(46–60)^b^	52(47–63)	54(45–61)
Non-responders	55(51–62)	56(50–60)	55(50–63)	58(52–60)
PPV (%)				
Responders	9(8–11)	8(7–12)	9(8–10)	8(7–11)
Non-responders	9(8–12)	8(8–10)	9(8–11)	9(8–11)
CI (L/min/m^2^)				
Responders	3.5(3.1–4.3)	4.1(3.4–4.9)^b^	3.5(3.2–4.3)	4.5(3.9–5.2)^a b c^
Non-responders	3.7(3.5–4.2)	3.8(3.5–4.2)	3.6(3.5–4.2)	3.9(3.8–4.4)

Results are present as median (25-75th percentiles)

*HR* Heart rate, *CVP* Central venous pressure, *SBP* Systolic blood pressure, *DBP* Diastolic blood pressure, *PPV* Pulse pressure variation, *CI* Cardiac index

^a^*p*<0<.05, comparison between responders and non-responders

^b^*p*<0.05, comparison between Supine transfer and baseline 1 or Fluid challenge and baseline 2

^c^*p*<0.05, comparison between Supine transfer and fluid challenge

### Hemodynamic effect of supine transfer test

**Fig. 2:**
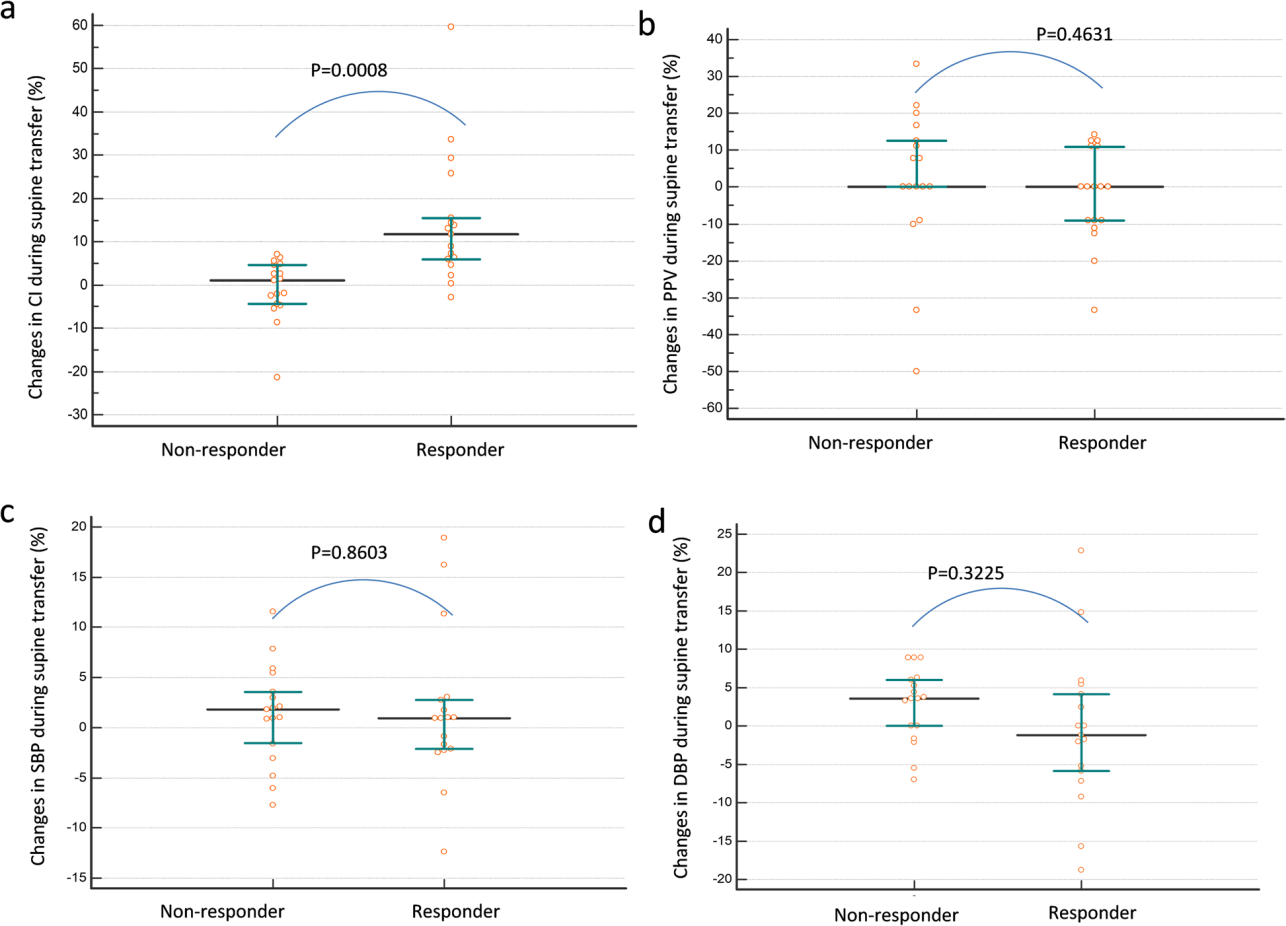
Changes in the cardiac index (CI) (**a**), pulse pressure variation (PPV) (**b**), systolic blood pressure (SBP) (**c**), and diastolic blood pressure (DBP) (**d**) during the supine transfer in non-responders and responders. Values are in percent changes. The black horizontal line is the median, while the upper and lower green lines represent the 25th and 75th percentile

**Fig. 3:**
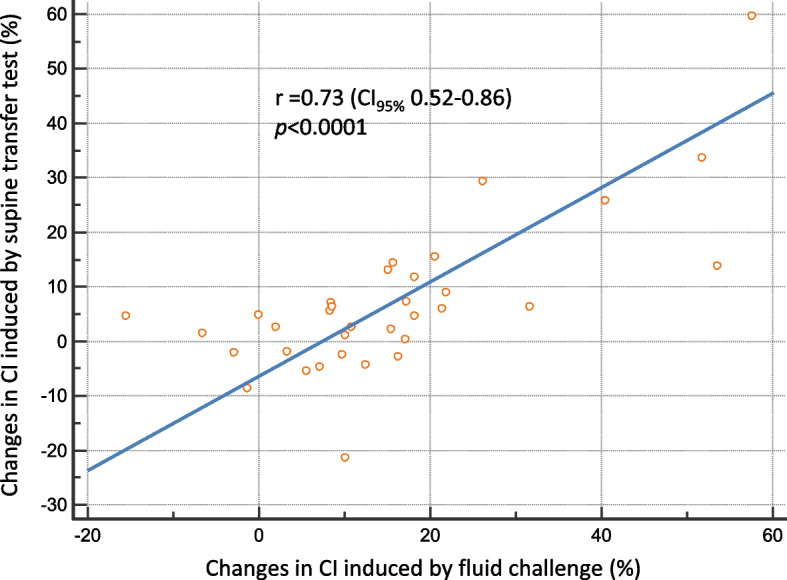
Liner correlation between the percentage changes in the cardiac index (CI) that was induced by the supine transfer test and fluid challenge. *CI*_*95%*_: 95% confidence interval

In all patients, the supine transfer test had a maximal effect on CI within 1 min, we wait for about 2 min. Hemodynamic parameters from baseline-1 to the supine transfer test are shown (Table 1, Fig. 1 and Additional file 1: Figure E3). The CI increased by 11.8% (from 3.5 (3.1–4.3) to 4.1 (3.4–4.9) L/min/m^2^; *p* = 0.0001) during the supine transfer test in responders, while no significant change was observed in non-responders (Table 1). The increase in CI that was associated with the supine transfer was significantly higher in responders than in non-responders (*p* = 0.0008; Fig. 2, Table 1). The percent changes in CI between the supine transfer test and fluid challenge were highly correlated, with an *r* value of 0.73 (Fig. 3).

### Hemodynamic effect of fluid challenge

The hemodynamic parameters from baseline-2 to the fluid challenge are shown in Table 1 and Fig. 1. After the fluid challenge, the CI increased by 20.7% (from 3.5 (3.2–4.3) to 4.5 (3.9–5.2) L/min/m^2^; *p* < 0.0001), while no significant change was observed in non-responders (Table 1). The increase in CI that was associated with the fluid challenge was significantly higher in responders than in non-responders (*p* = 0.0001; Table 1). Moreover, there was a significant difference in the CI between the supine transfer test and after the fluid challenge in fluid responders (Table 1). CVP and SBP were higher for fluid challenge than their baseline values.

### Ability of supine transfer to predict fluid responsiveness

**Fig. 4:**
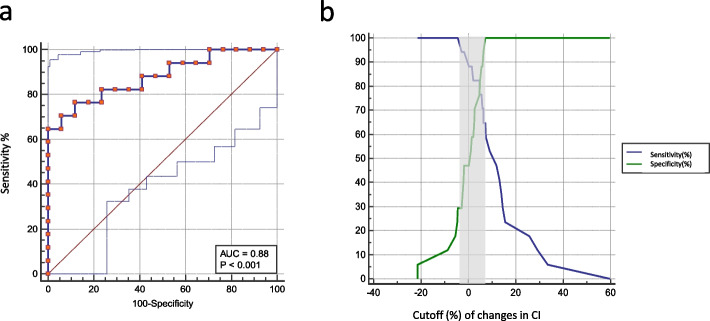
Receiver operating characteristic curve and gray zone analysis of the changes in the cardiac index (CI) (**a**, **b**) that were induced by the supine transfer test to predict fluid responsiveness. The blue dashed lines represent 95% confidence bounds

The AUC of the increase in CI that was induced by the supine transfer test to predict fluid responsiveness was 0.88 (95% confidence interval 0.72–0.97, *p* < 0.0001) (Fig. 4 and Table 2), and the increase in CI ≥ 5.5% predicted fluid challenge with 76.5% sensitivity (95% confidence interval 50.1–93.2%) and 88.2% specificity (95% confidence interval 63.6–98.5%) (Table 2). Nineteen (55%) patients fell within the gray zone (CI ranging from -3 and 8 L/min/m^2^) (Table 2 and Fig. 4b). The PPV, SBP, and DBP change during the supine transfer test did not accurately predict fluid challenge responsiveness (AUC 0.61 [0.43–0.78], *p* = 0.245; AUC 0.54 [0.43–0.78], *p* = 0.677; AUC 0.66 [0.48–0.82], *p* = 0.092) (Table 2 and Additional file 1: Figure E4).

**Table 2:** Predictive parameters of receiver operating characteristic (ROC) curves of variable percent changes induced by supine transfer

Parameters	ΔCI	ΔPPV	ΔSBP	ΔDBP
AUC (*CI*_*95%*_)	0.88 (0.72–0.97)	0.61 (0.43–0.78)	0.54 (0.36–0.71)	0.66(0.48–0.82)
*p*-value versus AUC = 0.50	< 0.01	0.25	0.678	0.09
Best cutoff value	5.5%	14%	1.7%	2.5%
Gray zone of optimal threshold	(-3% to 8%)	(-38% to 12%)	(-7% to 13%)	(-6% to 11%)
Patienst in gray zone, (number (%))	19(55%)	24(71%)	28(82%)	23(68%)
Sensitivity (CI_95%_)	76.5(50.1–93.2)	100(80.5–100.0)	70.6(44.0–89.7)	70.6 (44.0–89.7)
Specificity (CI_95%_)	88.2(63.6–98.5)	23.5(6.8–49.9)	52.9(27.8–77.0)	64.7 (38.3–85.8)
Positive predictive value (CI_95%_)	86.7(63.3–96.1)	56.7 (50.1–63.0)	60.0 (45.4–73.0)	66.7 (49.5–80.3)
Negative predictive value (CI_95%_)	78.9(61.0–90.0)	100. 0(-)	64.3 (43.2–81.0)	68.7 (49.3–83.3)
Positive likehood ratio (CI_95%_)	6.50(1.7–24.5)	1.31 (1.0–1.7)	1.50 (0.8–2.7)	2.00 (1.0–4.1)
Negative likehood ratio (CI_95%_)	0.27(0.1–0.6)	0.0 (-)	0.56 (0.2–1.3)	0.45 (0.2–1.0)
Youden index	0.65	0.24	0.24	0.35

*AUC* Area under ROC curve, *CI*_*95%*_ 95% confidence interval, *ΔCI* Percent changes in cardiac index, *ΔPPV* Percent changes in pulse pressure variation, *ΔSBP* Percent changes in systolic blood pressure, *ΔDBP* Percent changes diastolic blood pressure

## Discussion

This is the first study to evaluate the ability of the supine transfer test in detecting fluid challenges in patients with acute circulatory failure without intra-abdominal hypertension. In addition, results showed that an increase in CI greater than 5.5% during supine transfer test can predict fluid responsiveness with a gray zone between -3 and 8%.

The supine transfer test can be recommended for patients contraindicated to PLR. The supine transfer test transferred the blood from the splanchnic territory, as the “first part” of the PLR test. Compared with the PLR test, the supine transfer test involves lowering the trunk. Moreover, no special bed equipment is required [26]. The bed can lower the trunk and lift the legs simultaneously to maintain the angle of the trunk and legs at 135°, which is not accessible in most ICU wards. The supine transfer test can overcome some barriers, whereas PLR cannot. When patients have lower extremity disease (fractures, thrombosis, etc.) or conditions (wearing compression socks, etc.) that would decrease venous return to the heart chamber [8], the supine transfer test could be an alternative.

The present study showed that 55% of the patients fell into the gray zone, meaning that the data of only 45% of the patients were sufficiently accurate to guide fluid loading according to the changes in CI induced by the supine transfer test. A possible explanation for this finding may be that the supine transfer test causes smaller changes in the CI than the fluid challenge. However, the cut-off is also small (5%) in the tidal volume and mini-fluid challenges, which are popular tests for predicting fluid responsiveness; however, the percentage of patients included in the gray zone is approximately 14–20% [27]. Another possible explanation is that hemodynamic parameters undergo periodic changes due to the periodic variations in intra-pleural pressure during the respiratory cycle. Beat-to-beat variability is present in all hemodynamic parameters, which manifests in longer time intervals. The monitor has an update rate of 12 s. Spontaneous variability exists between each 12-s samples in all the parameters due to the underlying beat-to-beat variability. This spontaneous variability, such as in CI, could be within the same order of magnitude as the percentage changes in CI that were observed in this study during the supine transfer test for the responders (averaging 5.5%). This could possibly account for the wide range of the gray zone range and the high number of patients falling into the gray zone.

PPV is an accurate predictor in critically ill patients with mechanical ventilation, however, it is not applicable in various clinic scenarios [5]. Changes in PPV can detect preload responsiveness in patients with mechanical ventilated at < 8 ml/kg [28–30]. In the present study, we found that PPV or changes in PPV were not a reliable indicator in spontaneously breathing patients. Notably, only 17% patients in our study required mechanical ventilation. Patients with spontaneous breathing without positive pressure ventilation may experience small changes in cardiac loading condition. In a previous study, de Courson et al*.* conducted an evaluation of the least significant changes of PPV derived from pulse contour analysis, the results revealed that the least significant changes for PPV ranged from 10 to 4.9% [11]. These explanations may be able to clarify why PPV is an unreliable predictor in spontaneously breathing patients with pulse contour analysis.

To date, there is no easy and reliable method for predicting fluid responsiveness in patients with an elevated IAP. The PLR may lead to false-negative results [31, 32], which is not due to a decrease in intra-abdominal blood reserve, but might be secondary to a decrease in the recruitment of splanchnic or lower extremity blood towards the heart chambers due to intra-abdominal hypertension. The supine transfer test not only recruits splanchnic blood, but also lowers the IAP. It might be interesting to observe the changes in IAP and hemodynamic variables during the supine transfer test. This hypothesis must be confirmed by estimating the mean systemic pressure, CVP, and changes in IAP.

Our study has some limitations. When the supine position was changed, the joints of the body moved. Movement was restricted in patients with inconvenient hip joints. Second, joint rotation might provoke adrenergic stimulation and cause false positives; however, for the enrolled patients, the heart rate change was not significant; hence, the results were reliable. Third, this was a single-center study with a small sample size; further studies with larger sample sizes are required. Fourth, the supine transfer induced only small changes in the CI and an analysis of the least significant change was not conducted. Therefore, this limits the interest in this test and external validity of the results, as a low threshold value will limit the use of this test in patients under noninvasive hemodynamic monitoring, such as echocardiography, given the precision and variability of measurement with this device [33]. Fifth, it would have been interesting to perform subgroup analyses (mechanically ventilated vs. spontaneously breathing patients), which are necessary to clearly define the validity of the test. Unfortunately, not all these analyses were possible because of the study’s small sample size. Sixth, the study population was mainly a postoperative population compared with a normal ICU population. Patients in the surgical ICU had low rates of septic shock, norepinephrine infusion, and mechanical ventilation. In addition, 55% of the patients in this study fell into the gray zone; therefore, the supine transfer test should be used with caution when making clinical decisions. Lastly, ProAQT/Pulsioflex is an uncalibrated device used to measure the changes in CI, and its rliability in detecting small changes in hemodynamic parameters requires further investigation.

## Conclusion

The supine transfer test can potentially assist in detecting fluid responsiveness in patients with acute circulatory failure without intra-abdominal hypertension. Nevertheless, the small threshold and the 55% gray zone were noteworthy limitation.

### Ethics approval

The authors are accountable for all aspects of the work in ensuring that questions related to the accuracy or integrity of any part of the work are appropriately investigated and resolved. The study was conducted in accordance with the Delaration of Helsinki (as revised in 2013). The study (ChiCTR2200058264) was registered on 2022–04-04, and last refreshed on 2023–03-26. The study (No. 2203252–9-2303A, 2023.03.16) was approved by the ethics committees of Shanghai Cancer Center, Fudan University, China, and written informed consent was taken from all individual participants.

### Supplementary Information

**Additional file 1: Table E1.** Demographic and clinical information of included population. **Figure E1.** Study protocol. **Figure E2.** Study flowchart. **Figure E3.** Individual values of systolic blood pressure (SBP) (a, b), diastolic blood pressure (DBP) (c, d), and pulse pressure variation (PPV) (e, f) in each step of the responders and non-responders. **Figure E4.** Receiver operating characteristic curve and gray zone analysis of the changes in the pulse pressure variation (PPV) (a, b), systolic blood pressure (SBP) (c, d), and diastolic blood pressure (DBP) (e, f) that were induced by the supine transfer test to predict fluid responsiveness. The blue dashed lines represent 95% confidence bounds.

## Data Availability

All data generated or analyzed during this study are included in this article. The dataset used and/or analyzed during the study is available from the corresponding author on request.

## Abbreviations

ICU: Intensive care unit
PLR: Passive leg raising
CI: Cardiac index
IAP: Intra-abdominal pressure
V_T_: Tidal volumes
PEEP: Positive End-Expiratory Pressure
Pplat: Plateau Pressure
CVP: Central venous pressure
HR: Heart rate
SBP: Systolic arterial pressure
DBP: Diastolic arterial pressure
PPV: Pulse pressure variation
ROC: Receiver operating characteristic
AUC: Area under ROC curve
