# Supplementary material for: Supine transfer test-induced changes in cardiac index predict fluid responsiveness in patients without intra-abdominal hypertension
Source: BMC Anesthesiol. 2023 Sep 18;23:318. doi: 10.1186/s12871-023-02280-0 (PMC10506238; doi:10.1186/s12871-023-02280-0)
Supplement: Supplementary file 1 — Additional file 1: Table E1. Demographic and clinical information of included population. Figure E1. Study protocol. Figure E2. Study flowchart. Figure E3. Individual values of systolic blood pressure (SBP) (a, b), diastolic blood pressure (DBP) (c, d), and pulse pressure variation (PPV) (e, f) in each step of the responders and non-responders. Figure E4. Receiver operating characteristic curve and gray zone analysis of the changes in the pulse pressure variation (PPV) (a, b), systolic blood pressure (SBP) (c, d), and diastolic blood pressure (DBP) (e, f) that were induced by the supine transfer test to predict fluid responsiveness. The blue dashed lines represent 95% confidence bounds. [file 12871_2023_2280_MOESM1_ESM.docx]

**Electronic Supplementary Material**

| **ESM -Table E1. Demographic and clinical information of included population** | | | |
| --- | --- | --- | --- |
| **Variables** | **Entire cohort (n=34)** | **Responders (n=17)** | **Non-responders (n=17) P** |
| Age (year) | 62±11 | 61±12 | 63±10 0.32 |
| Male (*n*, %) | 24 (70) | 12 (70) | 12 (70) 0.73 |
| Hight (cm) | 166±6 | 167±7 | 166±6 0.65 |
| Weight (kg)  Admission categories: | 59±8 | 59±8 | 60±8 0.68 |
| Emergent surgery (*n*, %) |  |  | 0.52 |
| Laproscopic surgery-peritonitis | 2(6) | 2(12) | 0 |
| Planned surgery (*n*, %) |  |  | 0.32 |
| Laparotomy-gastric | 2(6) | 2(12) | 0 |
| Laparotomy-hepatic | 2(6) | 1(6) | 1(6) |
| Laparotomy-tumour debulking or whipple procedure | 5(14) | 3(17) | 2(12) |
| Laparotomy-colon | 4(12) | 1(6) | 3(17) |
| Laparotomy-gynaecology tumour sebulking | 3(9) | 1(6) | 2(12) |
| Orthopaedic resection of malignant neoplasm of bone | 5(14) | 3(17) | 2(12) |
| Urology nephrectomy | 2(6) | 1(6) | 1(6) |
| Medical (*n*, %) |  |  | 0.53 |
| Respiratory arrest | 1(3) | 1(6) | 0 |
| Pneumonia | 4(12) | 1(6) | 3(17) |
| Aspiration | 2(6) | 0 | 2(12) |
| Sepsis | 2(6) | 1(6) | 1(6) |
| Reason for enrollment: |  |  |  |
| Hypotension (*n*, %) | 10(29) | 7(41) | 3(17) 0.08 |
| Tachycardia (*n*, %) | 12(35) | 8(47) | 4(24) 0.09 |
| Skin mottling (*n*, %) | 3(9) | 1(6) | 2(12) 0.53 |
| Lactate > 2.0mmol/L (*n*, %) | 12(35) | 6(34) | 6(34) 0.62 |
| Oliguria(*n*, %) | 5(14) | 3(17) | 2(12) 0.48 |
| Vaspressor/inotropic support | 8(24) | 3(17) | 5(29) 0.07 |
| Apache Ⅱ score | 9(7-11) | 9(7-14) | 8(7-11) 0.28 |
| Lactate (mg/dL) | 1.7 (1.4-2.1) | 1.7 (1.3-2.1) | 1.6 (1.4-2.1) 0.15 |
| Death in the ICU (*n*, %) | 1 (3) | 1 (6) | 0 0.50 |
| Mechanical ventilation (*n*, %) | 6 (17) | 4 (24) | 2 (12) 0.09 |
| Tidal volume (predicted body weight, ml/kg) | 7.1(6.6-7.6) | 6.8(6.7-7.0) | 7.3(7.2-7.4) 0.54 |
| Positive end-expiratory pressure (cmH_2_O) | 5(5-5) | 5(5-5) | 5(5-6) 0.71 |
| Plateau pressure (cmH_2_O) | 18(17-18) | 18(17-18) | 17.5(17-18) 0.12 |
| Norepinephrine dose (μg/kg/min) (in treated patients) | 0.30 (0.18-0.33) | 0.30(0.29-0.35) | 0.30(0.15-0.31) 0.54 |
| Medical history: |  |  | 0.12 |
| Congestive heart failure (*n*, %) | 3 (9) | 2 (12) | 1 (6) |
| Chronic respiratory insufficiency (*n*, %) | 2 (6) | 1 (6) | 1 (6) |
| Hypertension (*n*, %) | 16 (47) | 7 (41) | 9 (53) |
| Diabetes (*n*, %) | 7 (21) | 1 (6) | 6 (35) |
| Chronic renal failure (*n*, %) | 1 (3) | 0 | 1 (6) |
| Cause of circulatory failure: |  |  | 0.42 |
| Hypovolemic shock (*n*, %) | 21(62) | 11 (65) | 10 (59) |
| Cardiogenic shock (*n*, %) | 1 (3) | 0 | 1 (6) |
| Septic shock (*n*, %) | 11 (32) | 6 (35) | 5 (30) |
| Other causes (*n*, %) | 1 (3) | 1 (6) | 0 |
| *ICU* : Intensive Care Unit, results are present as mean ± standard deviation, number ( frequency in %) or median (25-75th percentiles). | | | |


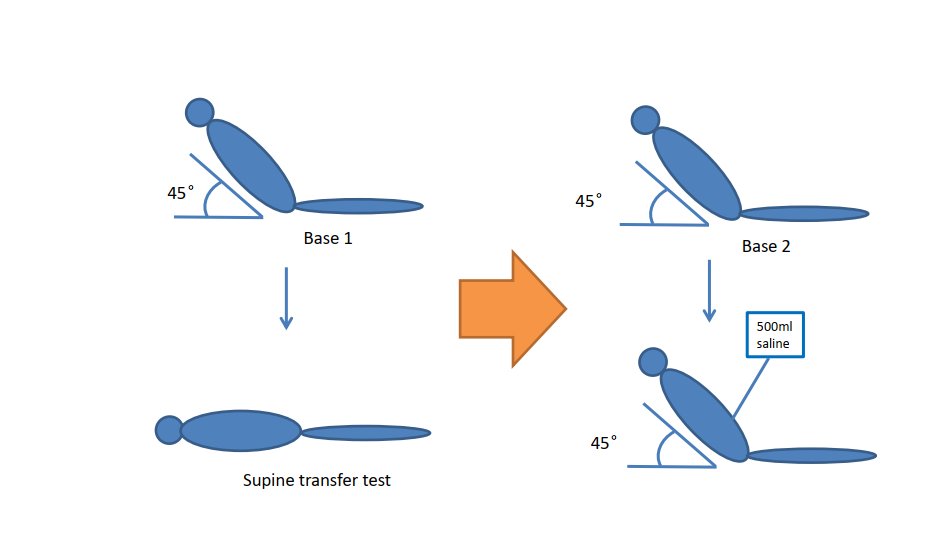


ESM- Figure E1: Study protocol.


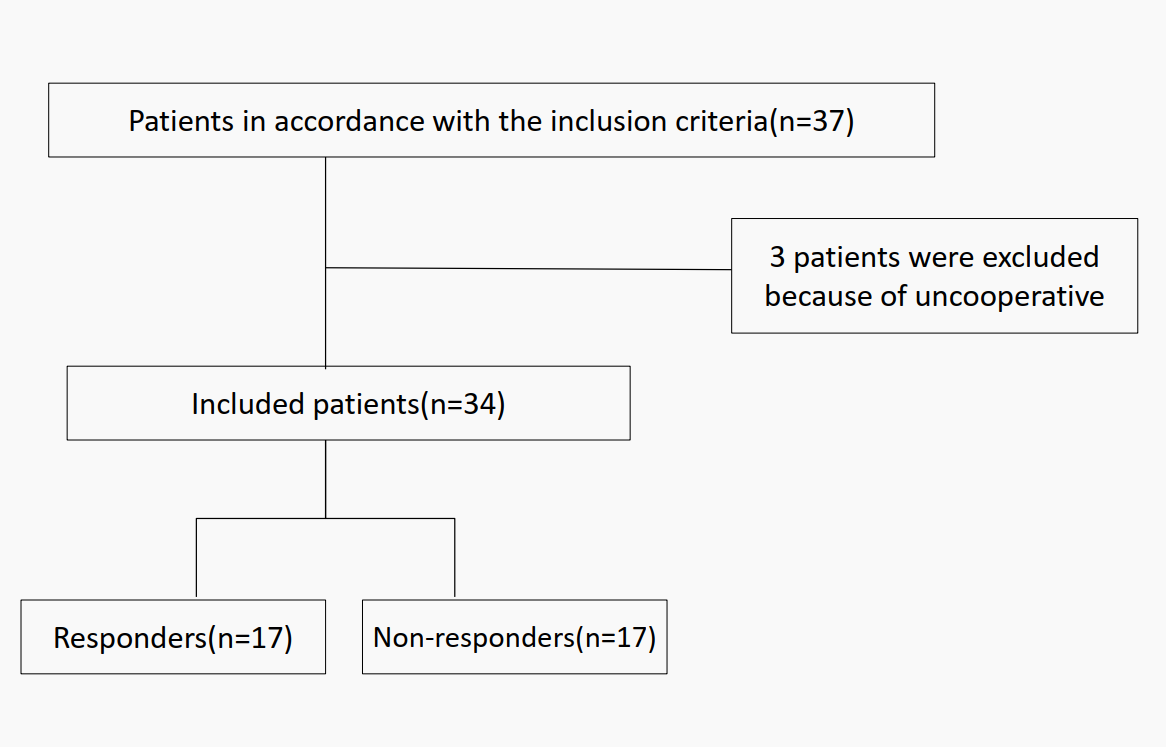


ESM-Figure E2. Study flowchart


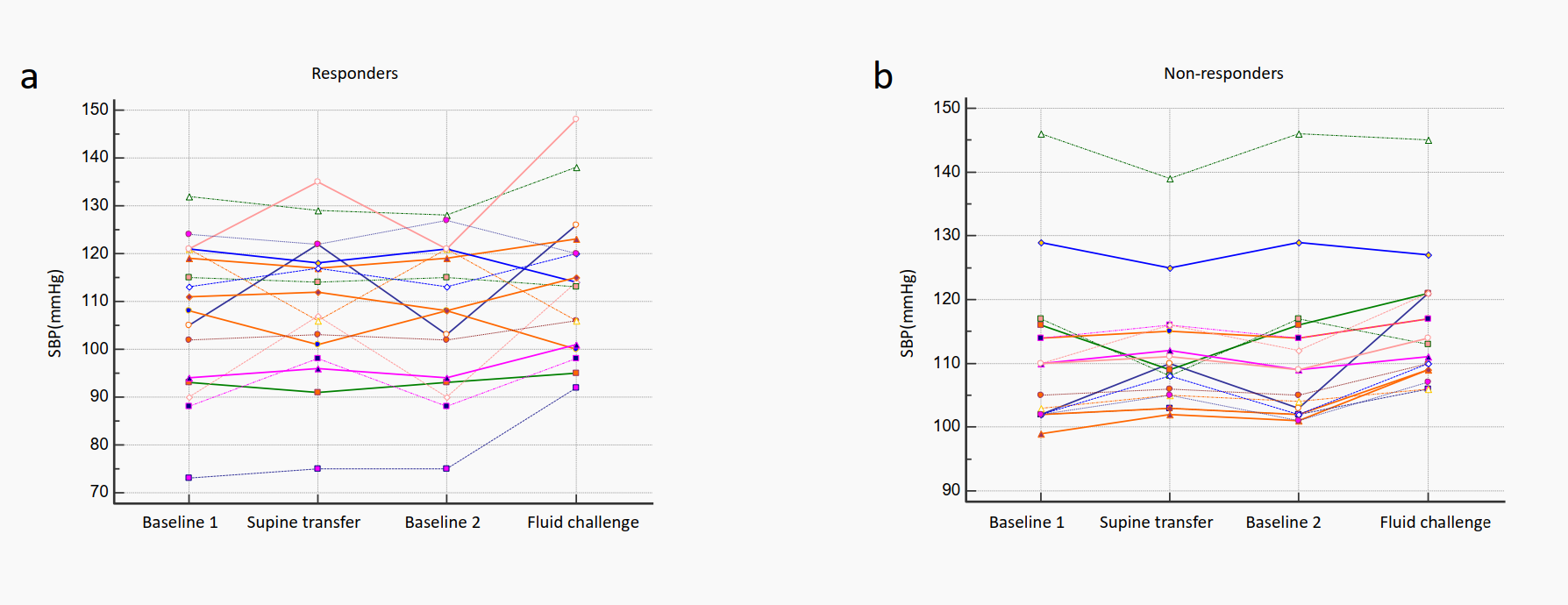


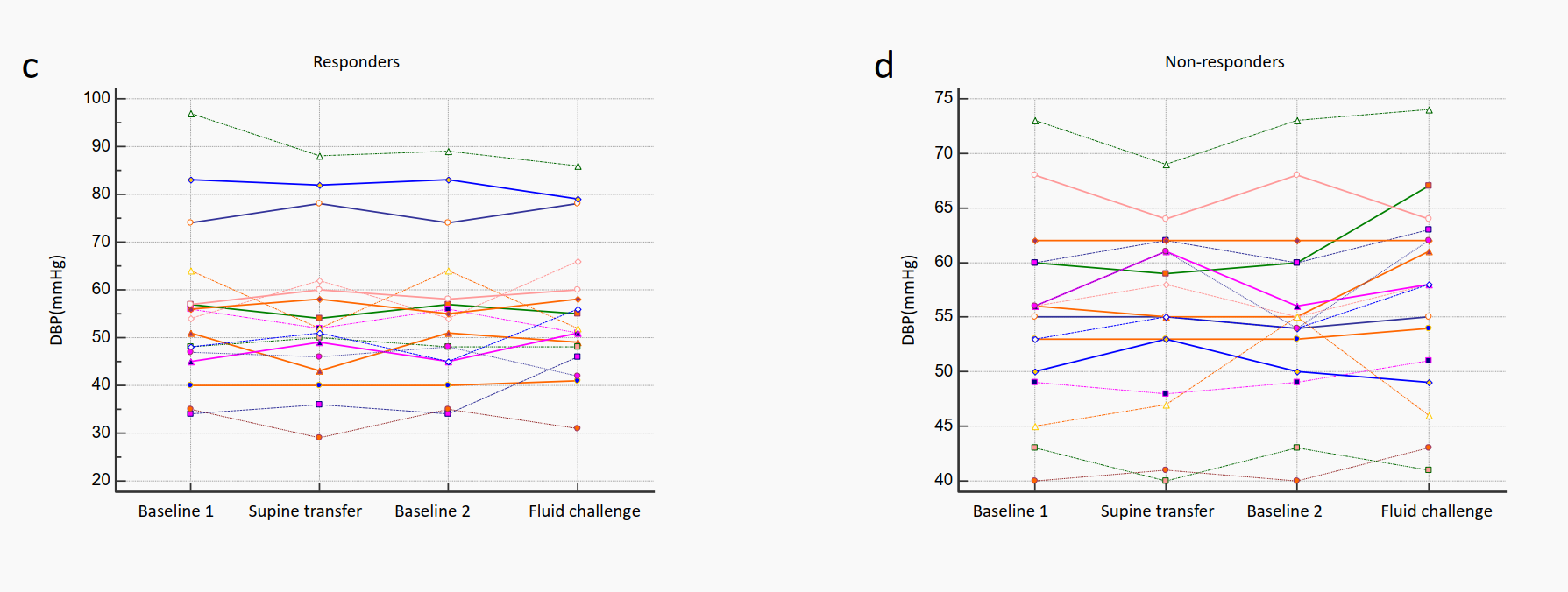


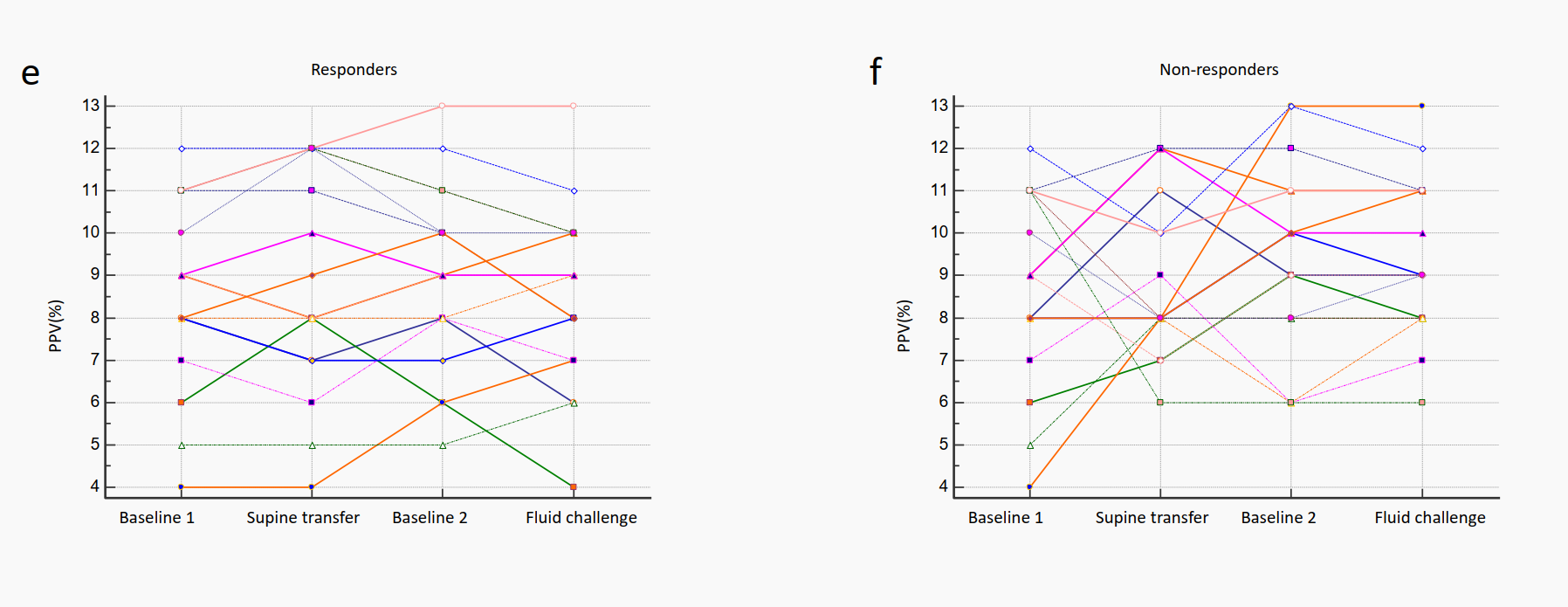


ESM- Figure E3. Individual values of systolic blood pressure (SBP) (a, b), diastolic blood pressure (DBP) (c, d), and pulse pressure variation (PPV) (e, f) in each step of the responders and non-responders.


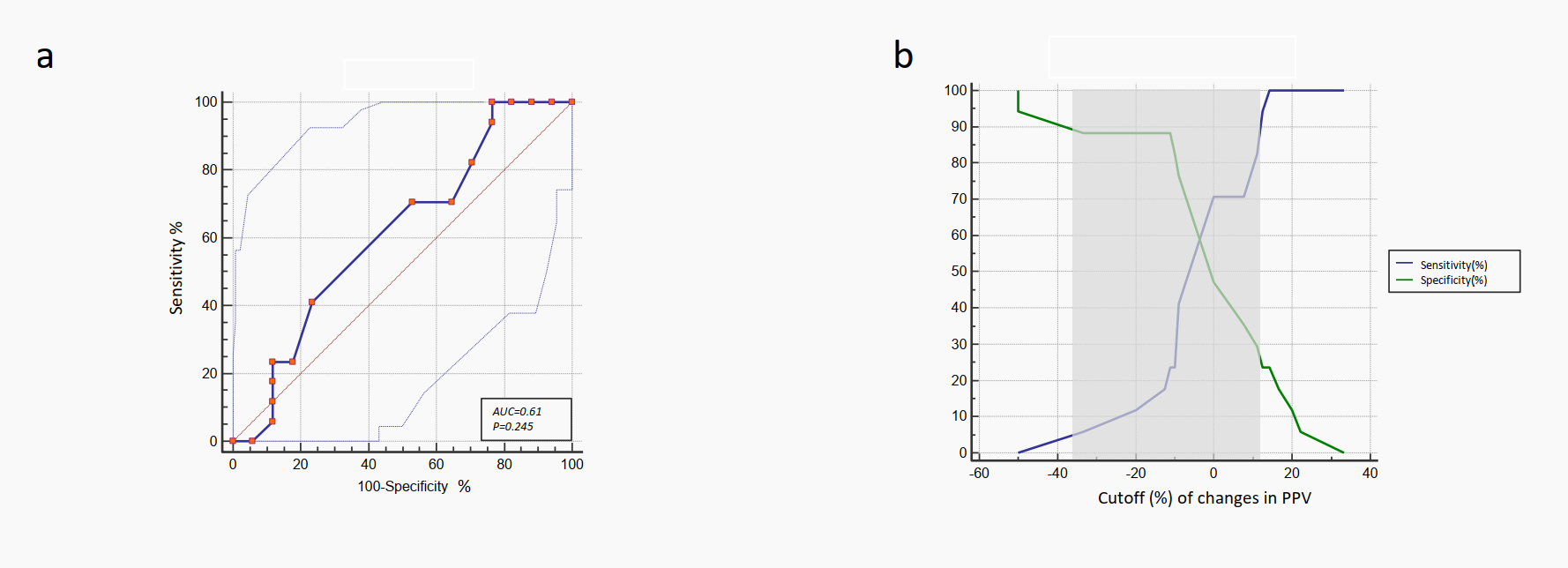


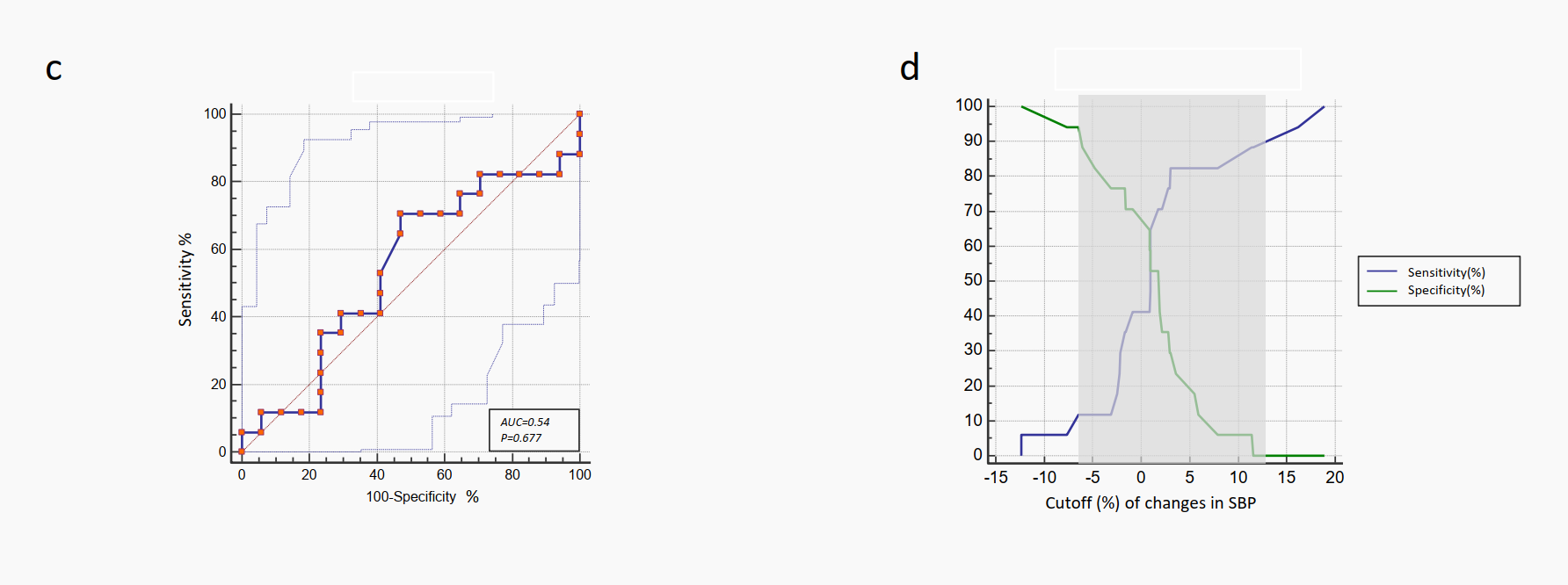


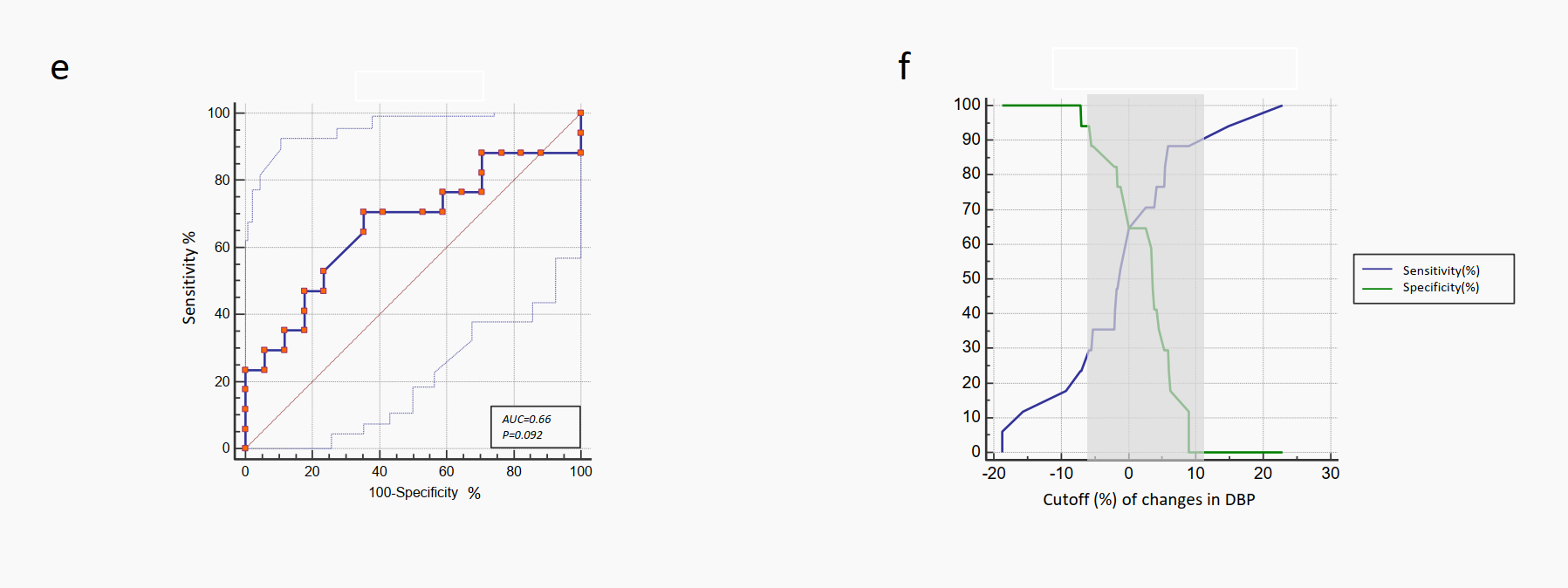


ESM-Figure E4. Receiver operating characteristic curve and gray zone analysis of the changes in the pulse pressure variation (PPV) (a, b), systolic blood pressure (SBP) (c, d), and diastolic blood pressure (DBP) (e, f) that were induced by the supine transfer test to predict fluid responsiveness. The blue dashed lines represent 95% confidence bounds.
